# A rare case of fulminant type 1 diabetes mellitus accompanied by both acute pancreatitis and myocarditis - case report

**DOI:** 10.1186/s12902-020-00607-3

**Published:** 2020-08-18

**Authors:** Fujiko Egashira, Midori Kawashima, Ai Morikawa, Minami Kosuda, Hisamitsu Ishihara, Kentaro Watanabe

**Affiliations:** Division of Diabetes and Metabolic Diseases, Department of Internal Medicine, Nihon University School of Medicine, 30-1 Oyaguchikami-cho, Itabashi-ku, Tokyo, 173-8610 Japan

**Keywords:** Fulminant type 1 diabetes mellitus, Pancreatitis, Myocarditis, Case report

## Abstract

**Background:**

Fulminant type 1 diabetes mellitus (FT1D) is a newly established subtype of type 1 diabetes. Its etiology has not been fully elucidated. Several cases with FT1D have exhibited pancreatitis or myocarditis.

**Case presentation:**

We report a 31-year-old Japanese woman who showed upper abdominal pain and was admitted to a local hospital. She was initially diagnosed with acute pancreatitis based on serum amylase elevation and swelling of the pancreas on computed tomography. Four days after admission, she developed diabetic ketoacidosis and was transferred to our hospital. Her symptoms and laboratory findings met the FT1D criteria. On the 3rd hospital day, electrocardiography (ECG) showed ST-segment elevation, and serum cardiac enzymes were markedly elevated. Because she exhibited late gadolinium enhancement in the apical wall on contrast-enhanced cardiac magnetic resonance imaging, she was diagnosed as acute myocarditis. Abnormal ECG findings and elevations of biomarkers associated with myocarditis showed improvement on the next day.

**Conclusions:**

This is the first case of FT1D accompanied by both pancreatitis and myocarditis and suggests that the pathophysiology of FT1D is related to the common etiology of acute pancreatitis and myocarditis.

## Background

Fulminant type 1 diabetes mellitus (FT1D) is a newly established subtype of type 1 diabetes. These specific clinical findings are shown at the onset of FT1D: 1) Hyperglycemia with diabetic ketoacidosis, 2) Plasma glucose level is greater than 16.0 mmol/L (288 mg/dL), whereas glycated hemoglobin level is less than 8.7%, 3) Urinary C-peptide excretion is less than 10 μg/day, while serum C-peptide level is less than 0.3 ng/mL (0.10 nmol/L) at overnight fast, or 0.5 ng/mL (0.17 nmol/L) following intravenous glucagon (or after a meal) load [1, 2]. Other important findings in FT1D are islet-related autoantibodies, such as antibodies to glutamic acid decarboxylase (GAD), islet-associated antigen 2 (IA-2) and insulin, generally being undetectable. However, the etiology of FT1D remains elusive. Several cases with concomitant acute pancreatitis [3, 4] or myocarditis [5–8] during the clinical course of FT1D were previously reported. Herein, we describe a 31-year-old Japanese woman with FT1D who was initially diagnosed with acute pancreatitis and then developed diabetic ketoacidosis and myocarditis. To our knowledge, there have been no prior reports of FITD associated with both pancreatitis and myocarditis.

## Case presentation

**Fig. 1:**
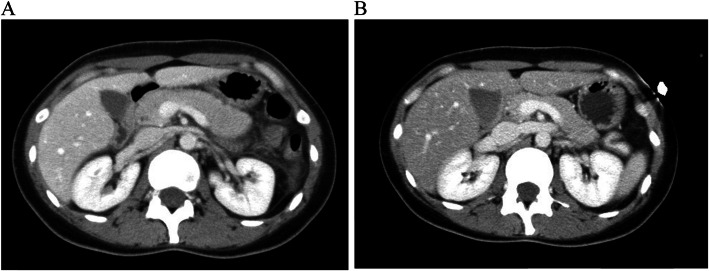
Abdominal CT scans. **a**: At the local hospital, imaging studies revealed swelling of the pancreas and mild ascites. **b**: On admission to our hospital, no abnormalities were detected. CT: computed tomography

A 31-year-old Japanese woman who presented with upper abdominal pain was admitted to a local hospital. She had a history of mycoplasma pneumonia in childhood and a family history of diabetes mellitus of her father. Four days before admission, she had a fever over 37 degrees and received an antipyretic agent at the local clinic, while flu testing results were negative. On admission to the local hospital, serum amylase was elevated to 370 IU/L, and abdominal computed tomography (CT) scanning showed pancreatic swelling and mild ascites (Fig. 1a). She was diagnosed with acute pancreatitis (grade 1) and treated with ulinastatin. The morning after admission, her fasting serum glucose, insulin and C-peptide level were within normal ranges: 101 mg/dL, 2.2 μIU/L and 0.8 ng/mL, respectively. At 4 days after admission, she complained of nausea and low back pain, and fell to consciousness disturbance (Japan Coma Scale II-10: drowsiness) with hyperglycemia (861 mg/dL) and metabolic acidosis. She was diagnosed with diabetic ketoacidosis and transferred to our hospital.

**Fig. 2:**
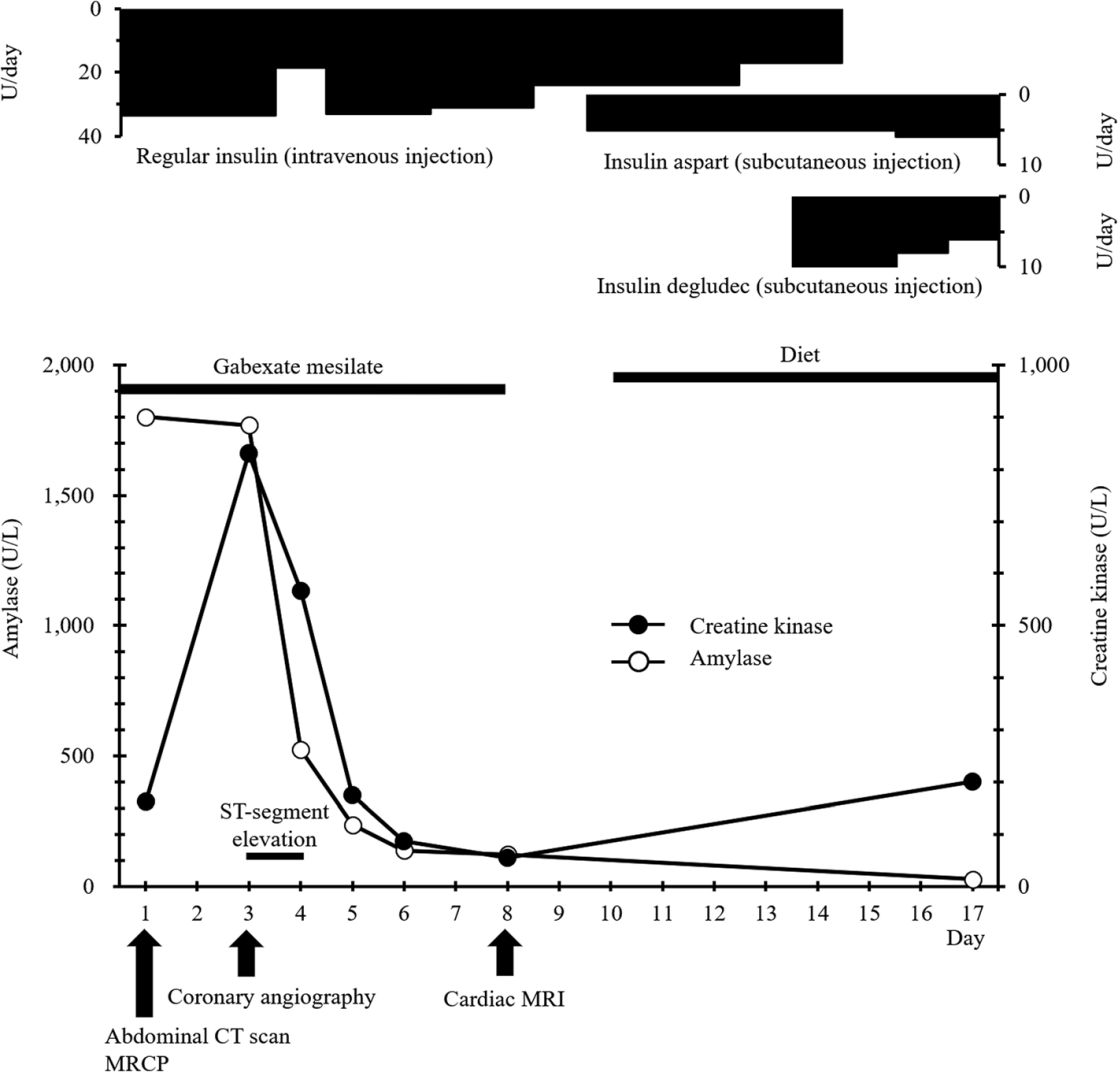
Clinical course during hospitalization.: magnetic resonance cholangiopancreatography, MRI: magnetic resonance imaging

This patient’s clinical course in our hospital is presented in Fig. 2. On admission to our hospital, she showed confusion (Japan Coma Scale I-2) and the upper abdominal symptom of nausea. Laboratory findings showed metabolic acidosis (pH, HCO_3_, and the anion gap in arterial blood were 7.07, 2.7 mmol/L, and 27.7 mmol/L, respectively), hyperketonemia (serum total ketone bodies 13,179 μmol/L) and hyperglycemia (612 mg/dL). Hemoglobin A1c (5.7%) was within the normal range, and anti-GAD and anti-IA-2 antibodies were undetectable. In addition, neither her serum C-peptide after a glucagon test nor 24-h urinary C-peptide excretion was detectable. These findings met the criteria for FT1D with diabetic ketoacidosis, and the patient was immediately given intravenous insulin infusion therapy. Human leukocyte antigen (HLA) typing indicated DRB1*0401-DQB1*0301, and DRB1*1302-DQB10604 class II gene.

**Fig. 3:**
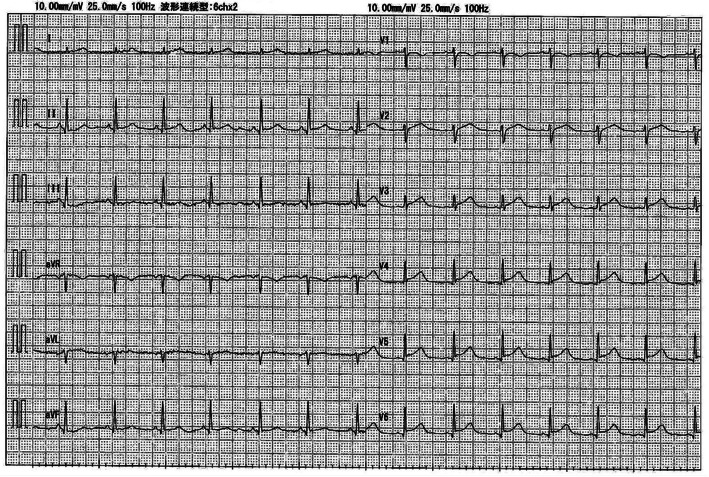
ECG the day after admission. ST-segment elevation was observed in V3-V6

**Fig. 4:**
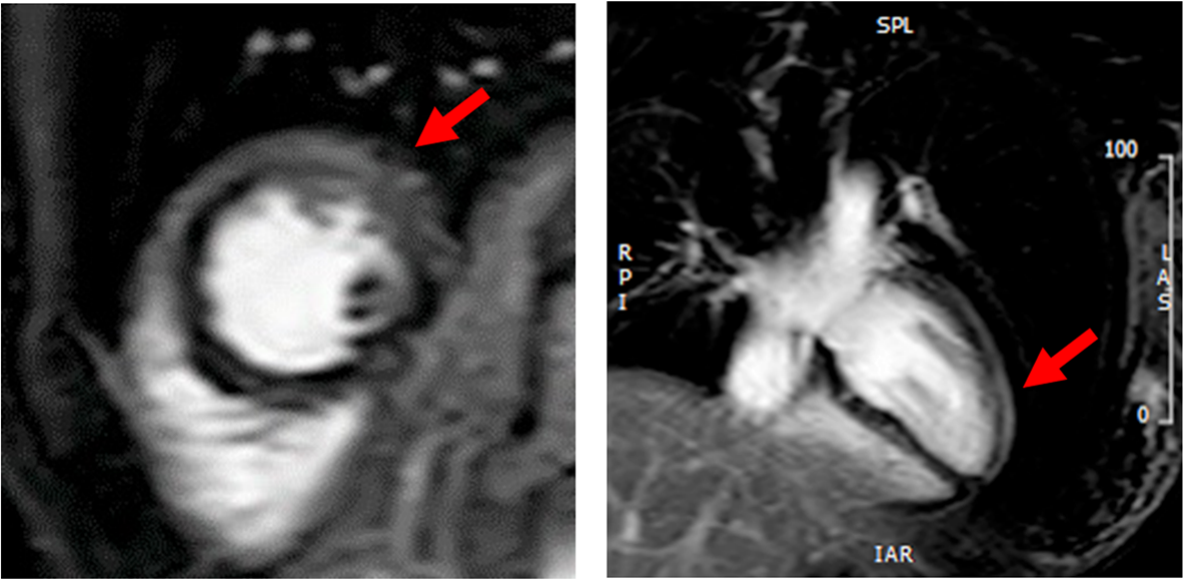
Cardiac MRI. LGE was observed in the apical wall (indicated by filled arrow). LGE: late gadolinium enhancement

Although serum amylase (1800 U/L, Fig. 2), lipase and trypsin (320 U/L and 3040 ng/mL respectively) were elevated, reexamination with CT scanning (Fig. 1b) showed amelioration of the pancreatic swelling and magnetic resonance cholangiopancreatography (MRCP) imaging depicted no abnormalities, even in the pancreas (data not shown). On the second day in our hospital, her symptoms showed improvement, and the serum glucose level had fallen below 200 mg/dL. However, on the 3rd hospital day, electrocardiography (ECG) depicted ST-segment elevation in V3-V6 (Fig. 3) without typical cardiac symptoms but serum creatinine kinase (CK) (830 U/L, Fig. 2), CK-MB and troponin I (49 U/L and 11.99 ng/mL respectively) were elevated. Coronary angiography showed no abnormal findings. Left ventriculography (LVG) showed apical wall hypokinesis, while no other abnormal findings, including takotsubo cardiomyopathy, were detected. On the 4th hospital day, abnormal ECG findings showed improvement and serum myocardial necrosis markers had normalized. On the 8th hospital day, contrast-enhanced cardiac magnetic resonance imaging (MRI) indicated late gadolinium enhancement (LGE) in the apical wall (Fig. 4). She was diagnosed with acute myocarditis based on the guidelines for diagnosis of myocarditis [9]. It should be noted that no abnormality was observed in echocardiogram examination and that serum CK level was 156 U/L at the previous hospital. On the 28th hospital day, follow-up cardiac MRI showed no LGE in the apical wall.

Because some viral infections are thought to cause FT1D, viral antibodies were measured at the time of admission and 4 weeks later: we tested for adenovirus, influenza A and B, echovirus 3, 7, 11, 12, coxsackie virus types A2, A4, A5, A6 A9, A16, B1, B2, B3, B4, B5, B6, parainfluenza virus 1, 2, 3, respiratory syncytial virus, Epstein-Barr virus, measles virus, mumps virus, herpes simplex virus, rubella virus, cytomegalovirus, and parvovirus. None of these tests revealed significantly elevated antibody titers. She was discharged on the 35th hospital day with intensive insulin treatment without medication for cardiovascular disorders or pancreatitis.

## Discussion and conclusions

We experienced a rare case of FT1D simultaneously affected by both acute pancreatitis and myocarditis. This case may be important for considering the etiology of FT1D, which might overlap with those of acute pancreatitis and myocarditis.

Elevations of exocrine pancreatic enzymes are commonly observed in FT1D and are listed among the findings, along with the main diagnostic criteria [2]. Indeed, some cases showing a definite association with acute pancreatitis have been reported [3, 4]. Several cases of FT1D also reportedly exhibited myocarditis [5–8]. To our knowledge, however, no prior reports on FT1D have documented a patient who showed acute pancreatitis and myocarditis within 1 week. Some cases showed the elevation of pancreatic exocrine enzymes with pancreatic swelling before the onset of FT1D [10]. Regarding myocarditis associated with FT1D, it has been reported that 4 cases developed myocarditis several days after symptoms of FT1D [5]. Our patient showed pancreatitis, FT1D and myocarditis consecutively in short period. Thus, it is reasonable to speculate that the same cause induced these three diseases.

What caused pancreatitis, the earliest symptom in this event? We presume that viral infection-induced pancreatitis because she had fever 4 days before the onset of acute pancreatitis and exhibited no common etiology of acute pancreatitis such as gallstones, biliary tract abnormality, excessive alcohol drinking, drug use and trauma. Dyslipidemia could cause pancreatitis if severe. However, we consider that the possibility is remote, since, although lipid data were not collected in the local hospital, serum triglyceride level was 251 mg/dL on admission to our hospital. About 10% of acute pancreatitis cases are caused by non-common miscellaneous factors such as infection with viruses, bacteria and parasites [11] with virus-induced pancreatitis being the leading etiology of infectious pancreatitis [12].

Viral infection, such as coxsackie viruses and cytomegaloviruse, are also considered to be involved in the development of FT1D [13]. Coxsackie virus infection can also cause pancreatitis and myocarditis [14–17]. Thus, it is tempting to hypothesize that our case had a coxsackie viral infection. In this regard, it is noteworthy that coxsackie and adenovirus receptor (CAR) expression levels are reportedly elevated in islets from type 1 diabetes and non-diabetes subjects with pancreatic islet cell autoantibodies, as compared with non-diabetic individuals [18]. However, the potential role of CAR expression in islets has yet to be determined.

Despite an extensive search for infections, however, no viral antibody elevations were detected in our present patient. We measured viral antibody titers in serum obtained immediately after admission (approximately 10 days after fever onset) and then again 1 month later. This timing may not be appropriate for detecting infections. Alternatively, an unknown virus may cause these pathological changes.

As another cause of FT1D in this case, the possibility of FT1D caused by hypersensitivity to drugs merits consideration. Fifteen cases of FT1D associated with the drug-induced hypersensitivity syndrome (DIHS) have been summarized [19]. Theoretically, ulinastatin used for treatment of pancreatitis in our case could cause FT1D, although this case met only 1 of 7 DIHS diagnostic criteria (data not shown).

In conclusion, we experienced a case with FT1D accompanied by both pancreatitis and myocarditis. To our knowledge, no prior case reports have described FT1D associated with concurrent pancreatitis and myocarditis. This case indicates that the development of FT1D may involve pathophysiological mechanisms in common with those of acute pancreatitis and myocarditis.

## Data Availability

The datasets used and/or analyzed during the current study are available from the corresponding author on reasonable request.

## Abbreviations

FT1D: Fulminant type 1 diabetes
ECG: Electrocardiography
GAD: Glutamic acid decarboxylase
IA-2: Islet-associated antigen 2
CT: Computed tomography
HLA: Human leukocyte antigen
MRCP: Magnetic resonance cholangiopancreatography
CK: Creatine kinase
LVG: Left ventriculography
MRI: Magnetic resonance imaging
LGE: Late gadolinium enhancement
CAR: Coxsackie and adenovirus receptor
DIHS: Drug-induced hypersensitivity syndrome
